# Treadmill-based submaximal VO_2_ estimation in patients with coronary artery disease: can a model derived from healthy individuals be valid?

**DOI:** 10.55730/1300-0144.6046

**Published:** 2025-06-23

**Authors:** Levent KARATAŞ, Esra Sena ORBAK YENİDÜNYA, Nesrin DEMİRSOY

**Affiliations:** Department of Physical Medicine and Rehabilitation, Faculty of Medicine, Gazi University, Ankara, Turkiye

**Keywords:** Cardiac rehabilitation, oxygen uptake, coronary artery disease, exercise, treadmill

## Abstract

**Background/aim:**

Existing treadmill-based VO_2_ prediction models may not accurately estimate submaximal VO_2_ in patients with coronary artery disease (CAD), as they are often derived from healthy populations. This study aimed to develop and validate a submaximal VO_2_ prediction model derived from healthy individuals and tested for generalizability in CAD patients by incorporating clinically relevant parameters.

**Materials and methods:**

A retrospective analysis was conducted with 101 participants (54 healthy, 47 CAD patients) undergoing cardiopulmonary exercise testing using the modified Bruce protocol. To better represent the submaximal VO_2_ reached during exercise, the average VO_2_ in the last minute of each stage was used. The model was developed using data from healthy individuals and subsequently validated in the CAD cohort. A linear mixed-effects model was employed to predict VO_2_ based on speed, grade, and other confounders, including peak VO_2_, body weight, and body mass index (BMI). The model’s performance was evaluated and compared with previously published equations using Bland–Altman plots, mean absolute error (MAE), root mean square error (RMSE), and Lin’s concordance correlation coefficient (CCC).

**Results:**

The final model, including speed, grade, and peak VO_2_, achieved an R^2^ of 0.83 (95% CI: 0.79, 0.86; f^2^ = 4.88). For CAD patients, the predicted-actual VO_2_ difference was −0.05 ± 1.8 mL/kg/min, with MAE and RMSE values of 1.4 and 1.8 mL/kg/min, respectively. The model outperformed reference equations, achieving the highest accuracy (CCC = 0.923) and minimal bias. Incorporating peak VO_2_ effectively accounted for exercise response differences between healthy individuals and CAD patients.

**Conclusion:**

A submaximal VO_2_ estimation model derived from healthy individuals and validated in CAD patients demonstrated high accuracy. Incorporating peak VO_2_ effectively bridged physiological differences, supporting individualized exercise prescriptions in cardiac rehabilitation. However, larger prospective cohorts are warranted to confirm external validity.

## 1. Introduction

Cardiac rehabilitation (CR) primarily revolves around aerobic training aimed at enhancing cardiorespiratory fitness (CRF) [1]. The intensity of aerobic exercise is determined by the amount of oxygen uptake (VO_2_) reached during activity. As exercise intensity increases, so does the VO_2_ level, with maximum VO_2_ (VO_2_max) influenced by factors such as age, sex, CRF, and medical conditions [2, 3]. Thus, aerobic exercise prescriptions are based on the intensity corresponding to the achievable VO_2_max [4].

Cardiopulmonary exercise testing (CPX) is considered the gold standard for determining VO_2_max and providing data essential for prognosis and exercise prescription [5, 6]. Therefore, in scenarios where direct VO_2_ measurement via CPX is not available due to the cost of such systems, VO_2_ estimation becomes vital [7].

Therapeutic exercise sessions in CR are designed as exercise bouts within a target VO_2_ range [8]. A practical approach to determining the treadmill speed and grade that achieves the target submaximal VO_2_ level is to use the patient’s CPX data as a guide. Although exercise intensity calculations are typically based on VO_2_max reserve, the achievement of the target during sessions is monitored using heart rate or perceived exertion levels [9]. However, various factors, such as medications, can confound heart rate-based intensity determination [10]. Furthermore, some patients may be unable to exercise at the same grade or speed as indicated by test protocol due to concurrent musculoskeletal issues [11]. In such cases, adjustments to speed and grade are necessary to achieve an exercise intensity at a comparable VO_2_ level [12].

The close relationship between VO_2_ and exercise workload is well-documented. While workload on cycle ergometers is expressed in watts, it is more difficult to define on treadmills due to the influence of multiple biomechanical factors such as speed and grade [13,14]. Lankford et al. have described the metabolic cost of walking across various speeds and extreme grades using polynomial equations [15]. Yet, the complexity of their formulas and the lack of validation in patient populations limit their clinical applicability. Practical equations aiming to estimate aerobic activity during a treadmill test generally focus on VO_2_max [16]. More than 40 years ago, the American College of Sports Medicine (ACSM) proposed a simpler treadmill-based formula for VO_2_max estimation that relied on speed and grade [17]. Later studies revealed that the ACSM formula tends to overestimate VO_2_max in both healthy adults and individuals with cardiovascular disease [18–20]. Recent studies with extensive databases and various patient groups have proposed more accurate equations to estimate VO_2_max (see the document, Supplemental Table, summarizing estimation models). However, the validity of these formulas in prescribing submaximal exercise for CAD patients remains unconfirmed [16, 21, 22].

Treadmill test protocols for VO_2_ prediction vary widely. For example, the ACSM formula assumes a steady-state VO_2_max plateau during exercise, while ramp protocol formulas do not [16, 22]. Typically, steady-state submaximal VO_2_ is reached after continuous or interval exercise lasting over 3 min [23]. Therefore, models estimating VO_2_max may not accurately reflect the stable submaximal VO_2_ seen in cardiac rehabilitation sessions [24].

Given these uncertainties, there is a clear need for a treadmill-based equation that can predict submaximal VO_2_ in patients with CAD. Therefore, this study aimed to develop a treadmill-based model for estimating submaximal VO_2_ in CAD patients. We hypothesized that a speed- and grade-based formula, derived from apparently healthy individuals and adjusted for individual factors, would more accurately predict submaximal VO_2_ in CAD patients.

## 2. Materials and methods

### 2.1. Participants

This retrospective cross-sectional study was conducted on records from the Cardiopulmonary Rehabilitation Unit at our institution between January 2020 and September 2024. CPX reports, all performed by the same practitioner (L.K.) for various indications (e.g., educational purposes, investigation of dyspnea etiology, functional capacity assessment, rehabilitation program evaluation), were examined. Detailed medical information was obtained from patient records. Data from eligible participants were included. The inclusion criteria were as follows: (1) age over 18 years, (2) a confirmed diagnosis of CAD (such as angina pectoris, myocardial infarction, percutaneous coronary intervention, or coronary artery bypass grafting) for the patient group, (3) no known cardiopulmonary disease for the healthy group, (4) completion of CPX using the modified Bruce protocol, and (5) attainment of maximal effort, defined as meeting at least one of the following: a respiratory exchange ratio (RER) ≥ 1.1, heart rate exceeding 90% of the age-predicted maximum, or a perceived exertion level of ≥ 17 on the modified Borg scale. Exclusion criteria included: (1) presence of chronic pulmonary disease, (2) heart failure (ejection fraction ≤ 40%, NT-proBNP ≥ 400 pg/mL, resting dyspnea, jugular venous distension, presence of bilateral lung rales, or peripheral edema), moderate to severe valve disease, or uncontrolled arrhythmia, (3) cardiac pacemaker, (4) active malignancy, (5) premature test termination due to coronary ischemia or arrhythmia, and (6) missing data on CPX. The study was approved by the Gazi University Ethics Committee with a number 2024-1701, and conducted in accordance with the principles of the Declaration of Helsinki. The informed consent was waived due to its retrospective design.

### 2.2. Outcome measures

Demographic and clinical data, including age, sex, diagnosis, medication use, height (cm), body weight (kg), and body mass index (BMI), were recorded. Following flowmeter and gas analyzer calibration, a symptom-limited maximum CPX was performed using an ergospirometry device with a breath-by-breath gas analysis system (Quark CPET; Cosmed, Rome, Italy). The modified Bruce treadmill protocol (Pluto med; h/p/cosmos sports & medical GmbH, Nussdorf–Traunstein, Germany) was employed, allowing participants to lightly hold the treadmill handrails throughout the test to mitigate the risk of falls. In patients with CAD, tests were conducted while they were on their regular medications to reflect clinical rehabilitation conditions. To ensure steady-state conditions were captured, the mean VO_2_ from the final minute of each stage was recorded, along with the corresponding mean heart rate, speed (m/min), and grade (expressed as a decimal). Data from incomplete stages were excluded from the analysis. Peak VO_2_, peak respiratory exchange ratio (RER), oxygen uptake efficiency slope (OUES), ventilatory equivalent for carbon dioxide (VE/VCO_2_) slope, as well as resting and peak heart rate and blood pressure values, were also documented. Since the tests were symptom-limited and many patients were unable to demonstrate a true steady-state VO_2_ plateau at maximal effort, we referred to the highest measured oxygen uptake as “peak VO_2_” rather than VO_2_ max in our analysis [23].

### 2.3. Statistical analysis

Nominal variables were presented as percentages, while continuous variables were expressed as means with standard deviations. The normality of continuous variables was assessed using histograms, the Kolmogorov–Smirnov test, and the Shapiro–Wilk test. Group comparisons between patients and healthy participants were performed using the chi-square test for nominal variables and the independent t-test for continuous variables. Pearson correlation analysis evaluated relationships between independent variables and VO_2_ levels at each stage.

The healthy participant group served as the training dataset for model development, while the patient group was used to test model validity. To predict VO_2_ levels at each stage, a mixed linear effects model for repeated measurements was conducted. Between-participant variance was treated as a random effect. In accordance with prior research, speed and the speed × grade interaction were included as primary independent variables in the fixed-effects component [16, 22]. Additional parameters showing significant correlations with exercise VO_2_ and distinguishing healthy participants from patients were incorporated into the model. The most explanatory model was identified based on the statistical significance of variable contributions, with model fit and parsimony evaluated using the Akaike Information Criterion (AIC) and Bayesian Information Criterion (BIC). To assess model generalizability and reduce overfitting risk, 10-fold cross-validation and 1000-iteration bootstrap resampling were also performed.

Model validity in the patient group was assessed using the mean absolute error (MAE), root mean square error (RMSE), one-sample t-test for predicted–actual VO_2_ differences, Bland–Altman plot analysis, and Lin’s concordance correlation coefficient (Lin’s CCC). A Lin’s CCC value ≥ 0.90 indicated sufficient agreement [25]. Proportional bias was analyzed using linear regression. Additionally, in the patient group, estimated VO_2_ values were computed using the ACSM walking equation, the Fitness Registry and the Importance of Exercise National Database (FRIEND) equation, the FRIEND heart failure (FRIEND-HF) equation, and the Fitness and Arthritis in Seniors Trial (FAST) equation (see the Supplemental Table for more information about the equations). The validity outcomes of the new model were compared against predictive performance of these equations. Given the retrospective nature of the study, the sample size was selected based on data availability, and a post hoc power analysis was employed to confirm the statistical strength of the model. Statistical analyses were conducted using SPSS version 26.0 (IBM Corp., Armonk, NY, USA) and R (v4.x; R Foundation for Statistical Computing, Vienna, Austria) with a p < 0.05 considered statistically significant.

## 3. Results

A total of 54 healthy individuals (27 males and 27 females) and 47 patients (30 males and 17 females) with CAD were included in the study. Demographic and clinical characteristics of participants are presented in Table 1. Compared to the healthy group, patients with CAD were older, had higher body weight and BMI, and exhibited lower peak VO_2_, RER, and OUES values. Additionally, their VE/VCO_2_ slope was significantly higher. The proportion of participants achieving maximal metabolic exertion, defined as RER ≥ 1.1, was 72% in the healthy group and 51% in the CAD group. When RER ≥ 1.05 was used as the threshold, these rates increased to 93% in the healthy group and 79% in the CAD group.

A total of 251 valid end-of-stage VO_2_ measurements were obtained from healthy participants, and 186 from patients. The relationships between potential predictors and end-of-stage VO_2_ in the healthy group are presented in Table 2. Correlation analyses revealed that speed, speed × grade, peak VO_2_, body weight, and BMI were associated with end-of-stage VO_2_. No significant differences in submaximal VO_2_ at each stage were observed between sexes (Table 3).

Based on these findings, the following variables were included in the linear mixed-effects model: speed, speed × grade, peak VO_2_, body weight, and BMI. The lower resting and maximum heart rates observed in CAD patients were likely due to the frequent use of beta blockers in this group. Since heart rate can be influenced by medications, it was excluded from the VO_2_ prediction model. The model development process is outlined in Table 4. The model with the lowest AIC and BIC values was selected as the best fit. The resulting VO_2_ prediction formula for exercise stages is as follows: VO_2_ = speed × (0.057 + 1.062 × grade) + 0.192 × peak VO_2_ + 3.106. The model demonstrated a strong fit, with an R^2^ value of 0.83 (95% CI: 0.79, 0.86) and a substantial effect size (f^2^ = 4.88). Additionally, the post hoc power analysis indicated a perfect power of 1.0. To address potential concerns regarding overfitting, we conducted a 10-fold cross-validation on the final model. The average root mean square error (RMSE) across folds was 2.55 (SD = 0.87), indicating stable and consistent predictive performance. Additionally, a bootstrap procedure with 1000 resamples was performed to evaluate the stability of the fixed-effect estimates. The 95% percentile confidence intervals for the model coefficients were as follows: intercept (1.06 to 5.26), speed (0.023 to 0.094), speed × grade interaction (0.94 to 1.19), and peak VO_2_ (0.14 to 0.25). These results support the robustness and generalizability of the model across the study population.

Using this model, predicted VO_2_ values were calculated for the patient group, revealing a small mean difference of −0.05 ± 1.8 mL/kg/min between predicted and actual VO_2_. MAE and RMSE were 1.4 mL/kg/min and 1.8 mL/kg/min, respectively. Table 5 summarizes the comparative validity analyses of our model in relation to other prediction equations applied to the patient group. The ACSM and FRIEND equations consistently overestimated VO_2_, while the FRIEND-HF equation tended to underestimate it. In contrast, the VO_2_ prediction obtained using the FAST equation yielded an average difference of −0.2 mL/kg/min from the actual VO_2_, which was not statistically significant.

**Figure 1:**
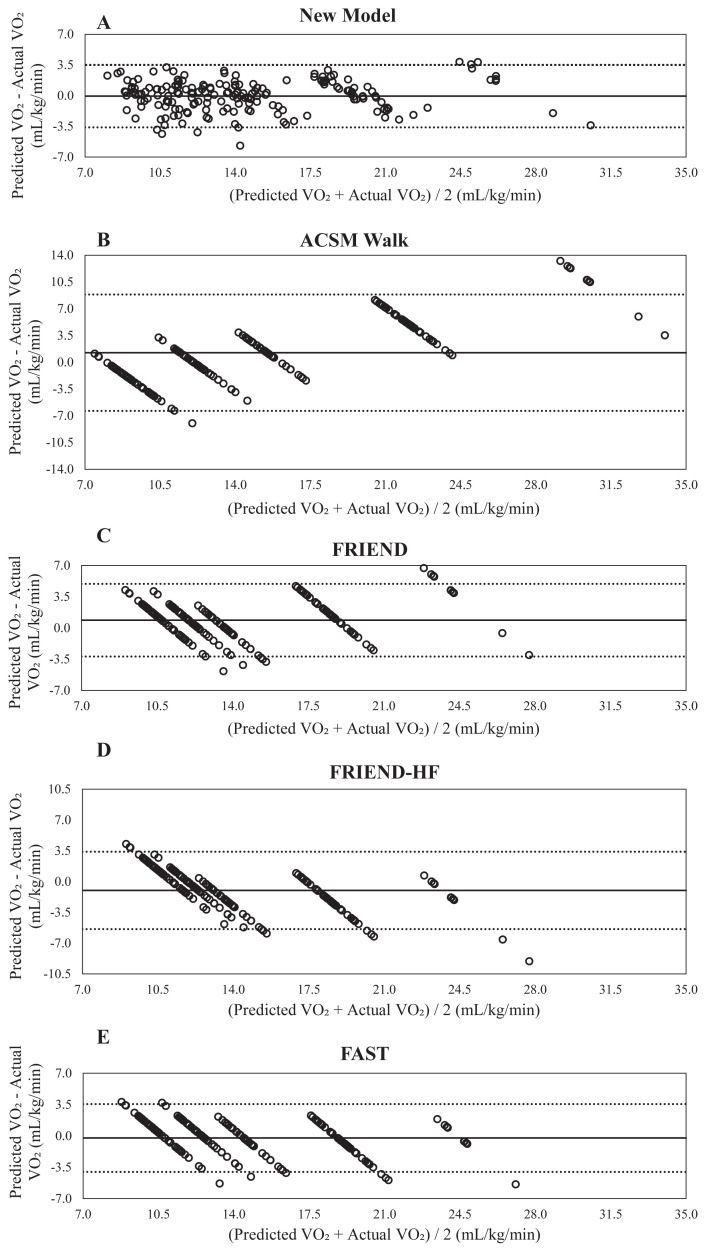
Bland-Altman plots of prediction models.

**Figure 2:**
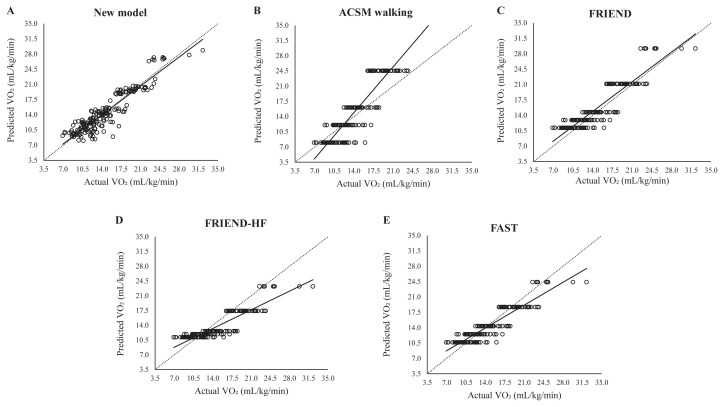
Dot plots representing the relationship between actual VO_2_ (x-axis) and predicted VO_2_ (y-axis) across prediction models.

Results from the Bland–Altman analysis, displayed in Figures 1 and 2, revealed that neither our study model (β = 0.08, p = 0.256) nor the FRIEND equation (β = 0.11, p = 0.117) exhibited any significant proportional bias. However, the ACSM equation demonstrated substantial proportional bias, underestimating VO_2_ at lower exercise intensities and overestimating VO_2_ at higher intensities (β = 0.788, p < 0.001). Both the FAST (β = −0.45, p < 0.001) and FRIEND-HF (β = −0.60, p < 0.001) equations exhibited proportional biases consistent with an underestimation of VO_2_ as exercise intensity increased.

Lin’s CCC results, reported in Table 5, showed that the new model exhibited a CCC of 0.923, indicating a strong level of concordance (≥ 0.90) between the predicted and observed VO_2_ values. In comparison, other prediction models demonstrated lower CCC values, suggesting less consistent agreement with the actual VO_2_ measurements.

## 4. Discussion

The findings of this study demonstrate that a model incorporating treadmill speed, grade, and peak VO_2_ provides a more accurate prediction of VO_2_ across varying exercise intensities in CAD patients compared to models based solely on speed and grade. The inclusion of peak VO_2_, which accounts for individual differences, has enabled our model—though initially developed with a healthy cohort—to achieve accurate VO_2_ predictions in CAD patients as well.

Previous studies have shown that VO_2_max estimation formulas, such as those recommended by the ACSM, may not yield sufficiently accurate results across diverse age groups within healthy adult populations [18]. This limitation has been attributed to the fact that ACSM formulas were originally based on data from young athletes. Consequently, the FRIEND equation, developed from a relatively large cohort of healthy adults, has been suggested due to its lower estimation errors [26]. However, research evaluating the validity of treadmill-based VO_2_max prediction models in CAD patients is limited. Jang et al. reported that in CAD patients, the FRIEND equation more accurately predicted CRF levels compared to the ACSM model [16]. Similar to our findings, the ACSM model overestimated VO_2_, particularly at higher exercise intensities in CAD patients. Their reported RMSE of 2.3 and Lin’s CCC of 0.870 are comparable to our results (RMSE = 2.4; Lin’s CCC = 0.888). However, Kokkinos et al. [22] found that the original FRIEND equation overestimated VO_2_max in heart failure patients with relatively lower cardiopulmonary fitness levels. As a result, the FRIEND-HF equation, which accounts for reduced speed and grade interaction effects, was recommended for heart failure populations. In line with these insights, our study found that variations in submaximal VO_2_ during different treadmill speeds and grades could be attributed to individual differences in peak VO_2_. Specifically, during submaximal exercise at the same speed and grade, patients with higher cardiopulmonary fitness tended to utilize more oxygen than those with lower fitness levels.

Among VO_2_ prediction models based on treadmill speed and grade, those incorporating a constant term, speed, and the speed × grade interaction have shown the highest accuracy [19]. In these models, the constant term reflects basal metabolism, the speed component represents the work done during horizontal walking, and the speed × grade interaction reflects work against gravity during graded walking. Our model incorporated the individual CRF, represented by peak VO_2_, as a corrective factor to these parameters. The results showed that our model had the lowest estimation and proportional bias errors, and it was the only model with a Lin’s CCC value above 0.90 in the CAD group, indicating greater accuracy compared to other equations. The FAST model, with parameters similar to ours, ranked second but exhibited proportional bias, underestimating VO_2_ at higher exercise intensities. This bias in the FAST model reduced its reliability in accurately predicting VO_2_ in patients with relatively high CRF. By including a peak VO_2_ component, our model overcame the potential issue of proportional bias, enabling its application across CAD patients with varying CRF levels. This result highlights that CRF is closely associated with the variability in submaximal VO_2_ achieved at similar exercise speeds and grades across individuals.

In the literature, the primary objective of prediction model development is often focused on calculating maximal VO_2_ to assess CRF [16]. However, limited evidence exists on the accuracy of these formulas in predicting submaximal VO_2_ levels, which are crucial for exercise prescription. Berry et al. suggested that their formula developed in the FAST cohort could predict VO_2_ levels at lower exercise stages as effectively as it does at peak VO_2_ [19]. However, the 2-min exercise stages used in their treadmill protocol may limit the ability of the FAST formula to accurately reflect the aerobic activity achieved during longer exercise sessions. Specifically, at higher speeds and grades, a time longer than 2 min is required to reach a steady-state VO_2_ [27]. This factor may explain the proportional bias observed with the FAST formula in our study, which used the 3-min stages in modified Bruce protocol.

A patient-tailored approach is essential for exercise prescription in CR. For example, shorter individuals or those with spinal stenosis may not tolerate high speeds, while patients with hip or knee problems may find high grades difficult to endure [11]. Therefore, in some cases, speed and grade must be adjusted to remain within the target VO_2_ range. However, the validity of conversion charts to achieve similar VO_2_ levels using different speed and grade combinations has primarily been demonstrated in high-speed running settings (e.g., 9.65–16 km/h) and lacks sufficient evidence for application in CR [12]. From this perspective, our new formula provides a foundation for adjusting treadmill speed and grade to determine exercise intensity in the rehabilitation of CAD patients. Although our study does not directly assess the impact of the model on exercise prescription outcomes, it introduces a physiologically meaningful approach by accounting for interindividual differences in submaximal oxygen consumption. In particular, it highlights that even under identical speed and grade conditions, oxygen uptake varies among patients, and this variability can be captured through the integration of peak VO_2_. Our model can serve as a practical guide for adjusting treadmill speed and grade interchangeably during exercise sessions, especially in patients who are unable to tolerate the initially prescribed speed or grade. This allows maintenance of a similar aerobic workload by targeting comparable submaximal VO_2_ levels, thereby supporting individualized and tolerable exercise prescriptions in CR.

Despite the valuable findings of our study, there are certain limitations due to its retrospective design. The sample size was relatively small; however, to increase statistical power, we included multiple measurements from each participant representing different exercise stages, resulting in a total of 251 observations. This, combined with the high post hoc power and additional resampling-based validation (10-fold cross-validation and bootstrapping), supports the robustness and reliability of the model.

Participants were permitted to use handrails during the CPX. While this could be viewed as a limitation in determining VO_2_ levels, it reflects the needs of many older patients with cardiovascular disease who may require handrail support during both exercise testing and sessions, thus enhancing the real-world applicability of the formula.

The speed and grade values analyzed were limited to those in the modified Bruce protocol, though the 3-min stages of the protocol provided a more accurate representation of VO_2_ scenarios encountered in exercise sessions. Although the model was tested in a separate group of patients with CAD, providing internal validation, independent external validation cohorts from different centers, various patient groups, or using alternate treadmill protocols were not available. This limits the generalizability of the model to broader clinical settings. Future studies will aim to externally validate the model in multicenter datasets and across different exercise testing protocols to confirm its wider applicability. Additionally, studies are needed to examine the validity of the model in larger cohorts divided into subgroups according to medical treatment regimens.

In conclusion, this study underscores the value of our equation in assessing aerobic activity during exercise in CAD patients by accounting for each patient’s individual CRF. Our proposed model demonstrated greater accuracy in predicting submaximal VO_2_ levels in CAD patients compared to traditional models based solely on speed and grade parameters. Additionally, the inclusion of a peak VO_2_ component reduced proportional bias, enhancing reliability across patients with varying functional capacities. These findings suggest that the model is a promising tool for calculating targeted submaximal VO_2_ levels to guide exercise intensity in clinical settings. However, further research is warranted to validate its applicability in other patient populations.

## Supplementary information

**Supplemental Table:** VO_2_ estimation models.

ACSM walking	VO _2_ = (0.1 × speed) + (1.8 × speed × grade) + 3.5
ACSM running	VO _2_ = (0.2 × speed) + (0.9 × speed × grade) + 3.5
FRIEND	VO _2_ = speed × (0.17 + 0.79 × grade) + 3.5
FRIEND-HF	VO _2_ = speed × (0.17 + 0.32 × grade) + 3.5
FAST	VO _2_ = (0.0698 × speed) + (0.8147 × speed × grade) + 7.533

Abbreviations: ACSM, American College of Sports Medicine; FAST, Fitness and Arthritis in Senior Trial; FRIEND, Fitness Registry and the Importance of Exercise National Database; FRIEND-HF, Fitness Registry and the Importance of Exercise National Database in Heart Failure.

VO_2_ is calculated in mL/kg/min. Speed is in m/min and grade is represented as a fraction.

## Figures and Tables

**Table 1:** Characteristics of participants.

	Healthy group (n = 54)	Patients group (n = 47)	p Value

Male/Female	27 (50)/27 (50)	30 (64)/17 (36)	0.227

Age, year	39.7 ± 9.5	56.2 ± 6.6	<0.001

Body weight, kg	74.6 ±15.4	81.7 ± 15	0.021

Height, cm	169.4 ± 8.6	169.1 ± 8.7	0.852

BMI, kg/m^2^	26 ± 4.9	28.5 ± 4.9	0.010

Referral diagnosis,			
Myocardial infarction	NA	19 (41)	NA
Coronary bypass surgery		10 (21)	
Percutaneous coronary intervention		16 (34)	
Stable angina pectoris		2 (4)	

Medications			
Beta-blockers	NA	41 (87)	NA
CCBs		9 (19)	
ACE inhibitors		18 (38)	
ARBs		14 (30)	
Coronary vasodilators		3 (6)	

Resting HR, beat/min	86 ± 14	79 ± 9	0.04

Peak HR, predicted max %	93.2 ± 8.5	87.7 ± 7.6	<0.001

Peak VO_2_, mL/kg/min	28.1 ± 5.5	21.3 ± 4.5	<0.001

Peak RER	1.15 ± 0.08	1.10 ± 0.09	0.007

OUES	2452 ± 595	2185 ± 525	0.019

VE/VCO_2_ slope	29.7 ± 4.5	31.7 ± 4.7	0.032

RPE in peak exercise	16.6 ± 2.8	17.5 ± 2.7	0.104

Highest stage completed in exercise test			
2. stage	0 (0)	5 (11)	<0.001
3. stage	0 (0)	2 (4)	
4. stage	22 (40)	30 (64)	
5. stage	29 (54)	10 (21)	
6. stage	3 (6)	0 (0)	

Abbreviations: ACE, angiotensin-converting enzyme; ARB, angiotensin receptor blocker; BMI, body mass index; CCB, calcium channel blocker; HR, heart rate; OUES, oxygen uptake efficiency slope; RER, respiratory exchange ratio; RPE, rate of perceived exertion; VE/VCO_2_, ventilatory equivalent for carbon dioxide; VO_2_, oxygen uptake. Data are presented as mean ± SD or n (%).

**Table 2:** Correlation analysis between potential predictors and end-of-stage VO_2_ in healthy individuals.

Predictors	Pearson Correlation (r)	95% CI	p Value
Speed × grade	0.895	0.867, 0.917	<0.001
Speed	0.861	0.825, 0.890	<0.001
Body mass index	−0.214	−0.329, −0.093	<0.001
Peak VO_2_	0.338	0.224, 0.443	<0.001
Body weight	−0.176	−0.293, −0.053	0.005
OUES	0.090	−0.034, 0.211	0.155
Age	−0.027	−0.150, 0.097	0.666
Peak RER	−0.024	−0.147, 0.100	0.701
VE/VCO_2_ slope	−0.021	−0.144, 0.103	0.742

Abbreviations: OUES, oxygen uptake efficiency slope; RER, respiratory exchange ratio; VE/VCO_2_ slope, ventilatory equivalent for carbon dioxide slope; VO_2_, oxygen uptake.

**Table 3:** Comparison of achieved submaximal VO_2_ at each stage between healthy males and females.

Submaximal VO_2_, mL/kg/min	Females	Males	p Value
1. stage	11.4 ± 3.5 n = 27	11 ± 2.3 n = 27	0.434
2. stage	13.2 ± 3.2 n = 27	13.2 ± 2 n = 27	0.973
3. stage	15.5 ± 2.6 n = 27	16 ± 1.6 n = 27	0.443
4. stage	20.8 ± 3 n = 27	22.2 ± 2.1 n = 27	0.052
5. stage	26.3 ± 4.3 n = 12	27.4 ± 3 n = 20	0.378

Abbreviations: VO_2_, oxygen uptake.

**Table 4:** Results of linear mixed model analysis.

Models	Explanatory variables	Estimates	p Value	AIC	BIC
Model 1	Intercept	5.8	0.026	1107.6	1114.6
	Speed	0.057	0.002		
	Speed × grade	1.063	<0.001		
	Peak VO_2_	0.170	0.004		
	Body weight	−0.027	0.486		
Model 2	Intercept	5.026	0.111	1106.5	1113.5
	Speed	0.057	0.002		
	Speed × grade	1.062	<0.001		
	Peak VO_2_	0.170	0.008		
	Body mass index	−0.050	0.486		
Model 3	Intercept	3.106	0.065	1103.5	1110.5
	Speed	0.057	0.002		
	Speed × grade	1.062	<0.001		
	Peak VO_2_	0.192	<0.001		

Abbreviations: AIC, Akaike’s Information Criterion; BIC, Schwarz’s Bayesian Criterion; VO_2_, oxygen uptake.

**Table 5:** Validation results of the VO_2_ prediction model in the patient group.

Models	Mean Difference^a^, ^b^, ^c^	MAE^a^, ^b^	RMSE^a^	Lin’s CCC
New model	−0.05 ± 1, p = 0.702	1.4 ± 1.1 95% CI: 1.2, 1.6	1.8 95% CI: 1.6, 2	0.923 95% CI: 0.898, 0.942
ACSM walking	1.25 ± 3.9, p < 0.001	3.0 ± 2.7 95% CI: 2.6, 3.4	4.1 95% CI: 3.5, 4.6	0.791 95% CI: 0.705, 0.824
FRIEND	0.9 ± 2.1, p < 0.001	1.8 ± 1.4 95% CI: 1.6, 2	2.4 95% CI: 2, 2.5	0.888 95% CI: 0.853, 0.915
FRIEND-HF	−1.0 ± 2.2, p < 0.001	1.9 ± 1.6 95% CI: 1.7, 2.1	2.5 95% CI: 2.2, 2.7	0.813 95% CI: 0.758, 0.857
FAST	−0.2 ± 1.9, p = 0.109	1.5 ± 1.3 95% CI: 1.3, 1.7	1.9 95% CI: 1.7, 2.2	0.890 95% CI: 0.856, 0.917

Abbreviations: ACSM, American College of Sports Medicine; CCC, concordance correlation coefficient; FAST, Fitness and Arthritis in Senior Trial; FRIEND, Fitness Registry and the Importance of Exercise National Database; FRIEND-HF, Fitness Registry and the Importance of Exercise National Database in Heart Failure; MAE, mean absolute difference; MD, mean difference; RMSE, root mean square error.

a in mL/kg/min;

b data are presented as mean ± SD;

c results of one sample t-test.
